# Bridging the training-practice gap in interprofessional student supervision

**DOI:** 10.1007/s40037-017-0330-8

**Published:** 2017-02-21

**Authors:** Priya Martin, Saravana Kumar, LuJuana Abernathy

**Affiliations:** 1 Allied Health Cunningham Centre, Darling Downs Hospital and Health Service, Toowoomba, Queensland Australia; 2 International Centre for Allied Health Evidence (iCAHE), School of Health Sciences, University of South Australia, Adelaide, Australia

**Keywords:** Interprofessional training, Clinical supervision, Student placements

## Abstract

**Background:**

Workforce recruitment and retention issues are common in highly dispersed regions such as Queensland in Australia. Provision of student placements in these non-metropolitan areas is one way of promoting staff recruitment. However, healthcare professionals in these areas face a number of challenges in accessing training opportunities including student supervision training. Funding was made available to develop and run a series of targeted, evidence-based, interprofessional student supervision workshops in non-metropolitan Queensland.

**Methods:**

Workshop participants were health professionals from both public and private health service providers in Queensland. Using a pre-post design, anonymous data were collected through surveys administered before and after workshop participation. Descriptive statistics were used to analyze participant information. Free text responses were categorized using an iterative process to identify prevalent themes.

**Results:**

A total of 147 participants attended nine face-to-face workshops and provided data. Allied health participants represented 70% of the population, with the remainder largely from nursing, medicine and dentistry. There was a positive shift in self-reported level of confidence in student supervision following training. Of the participants 143 (97%) reported that they acquired new skills and knowledge from training. A number of enablers of and barriers to translation of learning to practice following interprofessional student supervision training were identified.

**Conclusions:**

Targeted interprofessional student supervision training is valuable and can increase participants’ self-reported level of confidence in student supervision. It is recommended that health organizations promote a culture of providing positive student placement experiences in order to maximize future workforce opportunities.

## Introduction

Queensland is a geographically large, dispersed state in Australia where most people live in the metropolitan, south-east corner. Although many health professionals work outside large metropolitan centres, the number is substantially lower per capita compared with metropolitan areas [1]. There is a need to expand the workforce supply and the distribution of the health workforce to better meet community needs [2]. One way to address this is by increasing the student placement numbers in these areas as students with rural placement experiences have been shown to be more likely to return to rural practice following graduation [2]. To increase placement capacity, targeted supervisor development opportunities need to be made available as good clinicians do not automatically become good supervisors. Clear description of the supervisor role supported by education and training are ways to increase supervisor competence. Training has been shown to increase supervisor competency including teaching, assessment, appraisal, feedback and interpersonal skills [2]. However, much of the training for rural health practitioners is conducted in metropolitan locations or major centres. In addition to organizational and resource limitations, health practitioners in rural and remote settings also face geographical barriers in accessing training [3–5].

Although key elements of supervision of students are largely similar across various health professions, there are traditional barriers that deter health professionals coming together in the supervision arena [2]. However, evidence exists that interprofessional learning is a useful mechanism to maximize health professionals’ learning [6, 7]. Interprofessional delivery of supervision training is also well-suited to rural and remote practitioners as interprofessional practice is common in these areas.

The Cunningham Centre was contracted to deliver nine face-to-face interprofessional student supervision workshops primarily across non-metropolitan Queensland through funding from the Health Workforce Australia Clinical Supervision Support Program [8]. To address the gap in training opportunities in non-metropolitan Queensland, eight out of nine workshops were delivered outside larger, metropolitan centres. In this paper, we present the development, implementation and evaluation of an evidence-based interprofessional student supervision workshop and its impact on participants’ level of confidence in student supervision. We also explored participants’ intention to apply these learnings and identified the associated barriers and enablers within their clinical settings.

### The workshops

The workshops were developed to enhance participant knowledge and skills in student supervision. The one-day workshops were face-to-face, interprofessional, and evidence-informed with an optimal balance of theory and practice. The training was developed and delivered by a team of health professionals from occupational therapy, psychology, dietetics and nutrition, and podiatry. This ensured that perspectives from multiple professions were captured and utilized in training. Each workshop consisted of seven modules (Table 1).

**Table 1:** Outline of the workshop

Module One	Adult learning frameworks
Module Two	Models of student supervision
Module Three	Communication and feedback
Module Four	Reflective practice
Module Five	Effective supervision
Module Six	Managing challenging student behaviours
Module Seven	Considerations for rural and remote placements
Module Eight	Teaching styles

## Methods

### Design

The study used a pre-post design and collected data through a quantitative survey with free text response options. Data were collected at baseline before training and at post-baseline following workshop participation.

### Participants

Target participants were health professionals from both Queensland Health (the largest public health service provider in Queensland) and other non-governmental organizations. Participant recruitment was done through distribution of flyers and email advertisements through professional networks.

### Data collection

Anonymous data were collected from the baseline and post-baseline surveys (12 and 13 questions respectively). The baseline survey elicited quantitative and free text responses related to work and socio-demographics, self-rating of knowledge, skills and values related to student supervision. The post-baseline survey also elicited quantitative and free text responses about participant perceptions regarding the workshop, changes to their skills, knowledge and values related to student supervision. All data were reviewed for clarity and completeness, and entered by independent administrative support personnel.

### Data analysis

Descriptive statistics were used to analyze participant information. Free text responses were categorized and indexed using an iterative process used in analyzing formative data. Through this process prevalent themes were identified and their frequency determined.

### Ethical considerations

The study received exempt status from the Darling Downs Hospital and Health Service Human Research Ethics committee and adhered to professional and organizational standards of ethics requirements in research.

## Results

### Demographics

In total, 147 participants attended the workshops and all provided data. Participation at the workshops ranged from 13 to 22 participants. Of the participants, 70% (*n* = 102) were from allied health, 23% were from nursing, and the remainder were from medicine, dentistry and other health roles. A total of 43% (*n* = 63) of the participants reported undertaking clinical supervision training in the last two years.

### Level of confidence

**Fig. 1:**
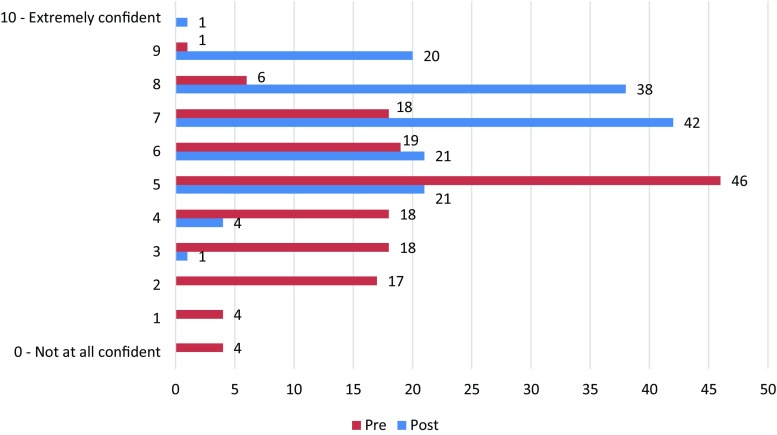
Pre- vs post-training level of confidence

Participants were asked about their level of confidence with student supervision at baseline before training and at post-baseline after training. There was a positive shift in the self-reported level of confidence in student supervision following workshop participation as shown in Fig. 1. Of the 147 participants, 143 (97%) reported that they acquired new skills and knowledge from training.

### Intention to apply learning in practice

Participants were asked about their intention to apply the knowledge and skills gained in their workplace following workshop participation; 98% (*n* = 144) of participants reported intention to make changes to their practice. Free text data from the post-baseline surveys were analyzed and summarized by two research team members. Themes that were derived included prioritizing time for supervision of students; increased confidence and motivation to supervise students; changing feedback practices to provide more timely and useful feedback; drive to engage in more effective supervisory practices (e. g., building positive supervisory relationships); and intention to use of tools, models and frameworks to structure activities within supervision (e. g., Gibb’s cycle to facilitate reflective practice; Proctor’s model to structure supervision sessions). When asked about their intention to apply the knowledge or skill in their workplace, one participant said:

‘… using the skills for more effective feedback and supervision, e. g., increase listening, increase awareness of learning styles, develop rapport and increase equality in the relationship’

Another participant while commenting on this said:

‘… structure placement better for students using models and student supervision. Give students more opportunity for feedback/reflective learning. Create clear expectations for students of what they should achieve on placement’

### Enablers and barriers

Participants were then asked about the factors in their workplace that would enable them to make the intended changes to practice. The top three responses were opportunity to use the skills gained (94.6%); quarantined time for student supervision (85.7%) and organizational support (81.6%). A further question focused on factors in the workplace that would hinder participants from making the intended changes. The top three barriers identified were competing priorities including service delivery pressures (81%), lack of opportunities to use the knowledge and skills gained (49.7%), and lack of organizational support (34.7%). When asked about the barriers, one participant said:

‘There is always growing pressure to deliver occasions of service and this impacts on dedicated time (for student supervision)’

While asked about enablers to apply learning in practice one participant stated:

‘Clinical support to compensate for time required to spend with student’

## Discussion

The purpose of this study was to evaluate the impact of evidence-based interprofessional student supervision workshops on participant level of confidence in providing supervision and to identify ways to bridge the training practice gap following training. Barriers and enablers reported in this study are consistent with those identified in previous studies and reviews [1, 2, 4]. Time is a key factor which can act both as an enabler of and a barrier to effective supervision [1, 4]. The findings of this study reinforce the importance of supervisors being able to quarantine and prioritize time for student supervision. This cannot be achieved without the support of the management and organization [2, 4]. When clinicians are supported to prioritize time for supervision, it promotes a positive supervision culture [1, 2].

There is an abundance of evidence in the literature that highlights the importance of a positive supervisory relationship [4, 6] which has been shown to have a direct effect on student self-efficacy and learning outcomes [2]. It is re-assuring that many participants understood the importance of a positive supervisory relationship and reported on intentions to build the supervisory relationships.

Although this study was undertaken in predominantly non-metropolitan settings, the findings are applicable to other contexts as student supervision practices are often similar across health professions and geographical contexts. Furthermore, enablers and barriers of translation of learning to practice post-training identified in this study are consistent with previous findings, from both metropolitan and non-metropolitan settings [1, 2, 4].

Post-baseline data were only collected at one point immediately post-training, which makes the long-term training effects unknown. Whilst this is a limitation, it is common practice in training evaluation as it captures responses from all participants with minimal opportunities for dropouts or non-respondents, and is feasible. Furthermore, given time and resource constraints, ongoing and long-term evaluation was not feasible. Future research in supervision training could address this.

## Conclusion

Targeted interprofessional student supervision training is valuable and can increase participants’ self-reported level of confidence in student supervision. A vast majority of participants reported the intention to make changes to supervision practice following workshop participation. A number of enablers and barriers related to making changes post-training have been identified. It is recommended that health organizations promote a culture of providing positive student placement experiences in order to maximize future workforce opportunities.
